# Microbiology testing associated with antibiotic dispensing in older community-dwelling adults

**DOI:** 10.1186/s12879-020-05029-z

**Published:** 2020-04-25

**Authors:** Zhuoxin Peng, Andrew Hayen, Martyn D. Kirk, Sallie Pearson, Allen C. Cheng, Bette Liu

**Affiliations:** 1 School of Public Health and Community Medicine, University of New South Wales (UNSW), Sydney, NSW 2052 Australia; 2 School of Public Health, Faculty of Health, University of Technology Sydney, Sydney, NSW Australia; 3 National Centre for Epidemiology and Population Health, Australian National University, Canberra, ACT Australia; 4 Medicines Policy Research Unit, Centre for Big Data Research in Health, University of New South Wales, Sydney, NSW Australia; 5 Menzies Centre for Health Policy, School of Public Health, The University of Sydney, Sydney, NSW Australia; 6 Department of Epidemiology and Infectious Diseases, Monash University and Alfred Health, Melbourne, VIC Australia

**Keywords:** Watch group antibiotics, Microbiology testing, Community, Stewardship

## Abstract

**Background:**

It is commonly recommended that microbiological assessment should accompany the use of antibiotics prone to resistance. We sought to estimate the rate of microbiology testing and compare this to dispensing of the World Health Organization classified “watch” group antibiotics in primary care.

**Methods:**

Data from a cohort of older adults (mean age 69 years) were linked to Australian national health insurance (Pharmaceutical Benefits Scheme & Medicare Benefits Schedule) records of community-based antibiotic dispensing and microbiology testing in 2015. Participant characteristics associated with greater watch group antibiotic dispensing and microbiology testing were estimated using adjusted incidence rate ratios (aIRR) and 95% confidence intervals (CI) in multivariable zero-inflated negative binomial regression models.

**Results:**

In 2015, among 244,299 participants, there were 63,306 watch group antibiotic prescriptions dispensed and 149,182 microbiology tests conducted; the incidence rate was 0.26 per person-year for watch group antibiotic dispensing and 0.62 for microbiology testing. Of those antibiotic prescriptions, only 19% were accompanied by microbiology testing within − 14 to + 7 days. After adjusting for socio-demographic factors and co-morbidities, individuals with chronic respiratory diseases were more likely to receive watch group antibiotics than those without, e.g. asthma (aIRR:1.59, 95%CI:1.52–1.66) and chronic obstructive pulmonary disease (COPD) (aIRR:2.71, 95%CI:2.48–2.95). However, the rate of microbiology testing was not comparably higher among them (with asthma aIRR:1.03, 95%CI:1.00–1.05; with COPD aIRR:1.00, 95%CI:0.94–1.06).

**Conclusions:**

Priority antibiotics with high resistance risk are commonly dispensed among community-dwelling older adults. The discord between the rate of microbiology testing and antibiotic dispensing in adults with chronic respiratory diseases suggests the potential for excessive empirical prescribing.

## Background

Antibiotic resistance is a severe threat to global public health. It is estimated that each year infections caused by antibiotic-resistant pathogens result in 700,000 deaths worldwide; the number might reach 10 million in 2050 if there is no effective action to curb resistance [1]. The overuse of antibiotics is considered as an important contributor to antibiotic resistance, [2] which could be effectively reduced by appropriate antibiotic stewardship [3]. To guide the use of antibiotics, the World Health Organization (WHO) proposed a three-category antibiotic classification system in 2017 [4]: namely access, watch, and reserve group antibiotics. Access group antibiotics are the first-line choices for common infections; watch group antibiotics are those with greater potential for developing resistance; and reserve group antibiotics are those considered “last resort” antibiotics for infections. WHO recommends that antibiotics in the watch and reserve groups (see Supplementary Table 1) should be limited to particular conditions and need special stewardship and monitoring [4]. There are also restricted antibiotic lists proposed in several countries for limiting the use of those antibiotics with high resistance potential [5–7]. Clinical guidelines for antibiotic prescribing in these countries also do not recommend them as the first choice for empirical therapy for common conditions in the community, e.g. respiratory tract infections, skin/wound infections, and urinary tract infections [8–10].

Earlier epidemiological studies and surveillance programs, nationwide and worldwide, have reported the use of some classes of watch/reserve group antibiotics and their relationship with antibiotic resistance in the population, e.g. the use of macrolides and *Streptococcus pneumoniae* resistance [11, 12], and the use of fluoroquinolones and cephalosporins and *Escherichia coli* resistance [11–13]. Some emerging multidrug-resistant organisms, including methicillin-resistant *Staphylococcus aureus* (MRSA) and vancomycin-resistant Enterococcus (VRE), were also reported to be associated with the use of quinolines and extended-spectrum cephalosporins [14, 15]. However, there are limited data describing the use of watch/reserve group antibiotics among different population subgroups, especially susceptible elderly people with major chronic diseases or living in Long-Term Care Facilities (LTCF), and comparing the rate of antibiotic dispensing with microbiology testing in primary care settings. Therefore, we examined the incidence rate of watch/reserve group antibiotic dispensing among community-dwelling older adults and compared it with the rate of microbiology testing for bacterial infections according to individual chronic health conditions in a large Australian cohort, in order to better understand the pattern of watch/reserve group antibiotic dispensing in general practice.

## Methods

### Study population and data sources

We used the Sax Institute’s 45 and Up Study, a large-scale cohort which recruited 267,153 participants aged ≥45 years from 2006 to 2009 in the largest Australian state, New South Wales (NSW). Participants were randomly sampled from the Department of Human Services (DHS) enrolment database. Detailed information on the cohort has been published previously [16]. Approximately 10% of adults aged 45 years and over in the general population of NSW were recruited. Participants completed a questionnaire at baseline about their demographics, lifestyle, and health information (available at https://www.saxinstitute.org.au/our-work/45-up-study/questionnaires/). They also agreed to have their questionnaire information linked to their health records. In this study, we linked the cohort to the Pharmaceutical Benefits Scheme (PBS), Medicare Benefits Schedule (MBS) based on a unique identifier provided by the DHS, and the NSW Admitted Patient Data Collection (APDC), and the NSW Registry of Births, Deaths and Marriages (RBDM) database through probabilistic linkage by the NSW Centre for Health Record Linkage (CHeReL).

The PBS database records medicines dispensed for outpatients subsidized by the Australian government pharmaceutical scheme; data include the dates of dispensing, medicine names, and WHO Anatomical Therapeutic Chemical (ATC) codes. About 98% of recorded antibiotic prescriptions in the PBS database were supplied from community pharmacies; others were supplied from private hospitals or other healthcare facilities [17]. The MBS database records general practitioner (GP) visits and other medical services provided to patients subsidized by the Australian government. The data include the types of service, the dates of service conducted, and the codes for service item (MBS item number). The APDC database records patient hospital admissions in NSW; data include the admission dates and diagnoses coded according to the International Classification of Diseases version 10 Australian Modification (ICD-10-AM). The RBDM database records registered death information, including the death dates of participants.

### Ethics

The study was approved by the University of New South Wales Human Research Ethics Committee (number 10186), and the NSW Population and Health Services Research Ethics Committee (HREC/10/CIPHS/97).

### Outcomes

Our primary outcome was the number of watch and reserve group systemic antibiotic prescriptions dispensed to each cohort participant recorded in the PBS database from 1st January 2015 to 31st December 2015 (observation period). The list of antibiotics classified as watch/reserve group is shown in Supplementary Table 1. We identified the antibiotic classes by their ATC codes [18].

A secondary outcome was the number of microbiology tests for bacterial infections received by each participant during 2015 based on records in the MBS database (see Supplementary Table 2). Some serology tests which might be used for diagnosing bacterial infections were also included. We excluded those microbiology tests only for viral, parasitic or fungal pathogens, and those tests provided in hospitals. As another secondary outcome, we examined the number of prescriptions of amoxicillin-clavulanate (ATC code J01CR02), the main broad-spectrum beta-lactam antibiotic outside the watch/reserve group dispensed in Australia during 2015 from the PBS database, since for many conditions prescriptions of this antibiotic would also require accompanying microbiology testing [10].

### Covariates

Socio-demographic factors for each participant were derived from the 45 and Up Study baseline questionnaire. We included sex (men or women), age-group in 2015 (45–59, 60–64, 65–69, 70–74, 75–79, and ≥ 80 years), education (university degree or higher, certificate or vocational education, no school certificate), annual household income at baseline in Australian dollars (low: <$30,000, middle: $30000- < $70,000, high: ≥$70,000), and residential area (major city, regional/remote area). Missing values in each covariate were included as a separate group.

The primary patient subgroups of interest in our study were those with major chronic diseases and those living in LTCF. We derived co-morbidities and other health service use from the MBS and APDC databases. For participants who were dispensed watch/reserve group antibiotics during 2015, an index date was defined as the first date of watch/reserve group antibiotic dispensing; for participants who were not provided watch/reserve group antibiotics, the index date would be 9th July 2015, the median date of watch/reserve group antibiotic dispensing among participants. Residence in a LTCF was ascertained if participants had an MBS record of a medical service in a LTCF (see Supplementary Table 2 for codes) in the year before the index date. A history of major chronic diseases was based on hospitalization records within 3 years before the index date in the NSW APDC database, defined by the primary diagnosis codes (ICD-10-AM): cancer (C00-C97), diabetes mellitus (E10-E14), chronic obstructive pulmonary disease (COPD, J40-J44), chronic kidney disease (N18), and cardiovascular diseases (i.e. ischemic heart diseases or stroke, I20-I25 or I60-I69). We also included self-reported asthma that was indicated in the baseline questionnaire. The number of GP consultations in the MBS database (see Supplementary Table 2 for codes) and the number of hospital admissions in the NSW APDC database in the year before the index date were included as covariates for each participant, as these factors were shown to be associated with antibiotic use in a previous study [19].

### Data analysis

In this analysis, we included participants who were alive on 1January 2015 based on death records in the NSW RBDM. Person-time in the analysis was calculated from 1 January 2015 to 31 December 2015 or date of death whichever came first. We calculated the number and incidence rate of watch/reserve group antibiotic prescriptions dispensing, microbiology testing, and amoxicillin-clavulanate dispensing in 2015 among the study population overall and according to different individual characteristics. Using multivariable zero-inflated negative binomial regression, we estimated adjusted incidence rate ratios (aIRR) and calculated 95% confidence intervals (CI) to identify the associations of major comorbidity and residence in LTCF with watch/reserve group antibiotic dispensing and microbiology testing. We also assessed risk factors for the dispensing of macrolides (in the WHO watch group but not in the Australian restricted antibiotics list [7]), other watch/reserve group antibiotics, and amoxicillin-clavulanate separately. To verify the potential effect of prior antibiotic use on watch/reserve group antibiotic dispensing, we performed a sensitivity analysis and only included those watch/reserve group antibiotics without antibiotic prescriptions in the 14 days prior. All the socio-demographic, co-morbidities, and health service-related covariates described above were included in the model.

We also examined the timing of microbiology tests in relation to the dispensing of watch/reserve group antibiotics. We calculated the proportion of watch/reserve group antibiotic prescriptions that had an accompanying microbiology test performed within 14 days prior to or 7 days after the dispensing date for study populations, considering that the tests performed around the day of dispensing are potentially related to the antibiotic treatment.

All analyses were conducted using Stata version 14.1. Two-sided *P* value< 0.05 was used as the threshold for statistical significance.

## Results

After excluding people who died before 2015, we included 244,299 individuals (mean age 69 years) of whom 120,747 (49%) were dispensed ≥1 antibiotic prescription in 2015. Among those 120,747 participants, a total of 403,492 antibiotic prescriptions were dispensed, of which 63,306 (16%) were watch/reserve group antibiotics. There were 29,917 (12%) participants who were dispensed at least one watch/reserve group antibiotic prescription: 13,914 (5.7%) were dispensed one; 10,102 (4.1%) were dispensed two; and 5901 (2.4%) were dispensed three or more prescriptions. There were fewer than five reserve group antibiotic prescriptions; thus, they were included in the watch group analyses; and in the following text, we simply refer to the group as watch group. As shown in Table 1, the most commonly dispensed watch group antibiotics were macrolides (53,336 prescriptions), followed by quinolones and fluoroquinolones (8519 prescriptions). Among non-watch group antibiotics, amoxicillin-clavulanate was dispensed in 67,735 (17%) prescriptions. Over the observation period, there were 149,182 microbiology tests for bacterial infections conducted in the study population. The distribution of the intervals between a dispensed watch group antibiotic prescription and its closest microbiology test is shown in Supplementary Figure 1, 69% of which were in the − 14 days to + 7 days window.

**Table 1:** Number of dispensed antibiotic prescriptions and microbiology tests by types

Antibiotic	No. (%)
**Total**	**403,492 (100)**
By type	
**Watch and reserve group**^**a, b**^	**63,306 (16)**
Macrolides (ATC code: J01FA)	53,336 (13)
Quinolones and fluoroquinolones (ATC code: J01M)	8519 (2.1)
Others ^c^	1451 (0.36)
**Antibiotics not in watch or reserve group**	**340,186 (84)**
Amoxicillin-clavulanate (ATC code: J01CR02)	67,735 (17)
Others	272,451 (68)
Microbiology test	No. (%)
**Total**	**149,182 (100)**
By type	
Urine examinations ^d^	82,291 (55)
Microscopy & culture for specimens of sputum	4887 (3.3)
Microscopy & culture for other specimens	34,014 (23)
Microbial antigens, nucleic acid, or antibody testing	24,432 (16)
Others	3558 (2.4)

^a^. As defined by the WHO Model List of Essential Medicines in 2017

^b^. Included reserve group antibiotics (*N* < 5)

^c^. Included 3rd&4th-generation cephalosporins (ATC code: J01DD, J01DE), glycopeptides (ATC code: J01XA), ticarcillin and a beta-lactamase inhibitor (ATC code: J01CR03)

^d^. Included microscopy and culture

In 2015, the incidence rate was 0.26 per person-year for watch group antibiotic dispensing and 0.62 per person-year for microbiology testing (Table 2). Overall, 11,993 (19%) watch group antibiotic prescriptions had a microbiology test within − 14/+ 7 days and were regarded as watch group prescriptions with a related microbiology test. Most patients with major chronic diseases or living in a LTCF had a higher incidence rate of watch group antibiotic dispensing, microbiology testing, and a higher proportion of antibiotic prescriptions with a related test when compared with other populations. However, patients with chronic respiratory diseases, i.e. COPD and asthma, were the exceptions, as only 17% of their prescriptions had a related microbiology test, lower than the rest of study populations.

**Table 2:** Number and incidence of dispensed watch group antibiotic prescriptions and microbiology tests by participants’ characteristics

Characteristic			Watch group antibiotic prescriptions			Microbiology tests	
	No. (%)	Person-years	No.	Incidence ^a^	No. (%) related to a test ^b^	No.	Incidence
Total	244,299 (100)	242,195	63,306	0.26	11,993 (19)	149,182	0.62
**Sex**							
Men	110,120 (45)	108,939	25,945	0.24	4908 (19)	58,787	0.54
Women	134,179 (55)	133,255	37,361	0.28	7085 (19)	90,395	0.68
**Age (years)**							
45–59	54,148 (22)	54,079	9874	0.18	1648 (17)	23,162	0.43
60–64	44,072 (18)	43,987	9824	0.22	1643 (17)	21,594	0.49
65–69	41,952 (17)	41,825	10,274	0.25	1794 (18)	22,786	0.55
70–74	35,584 (15)	35,394	10,423	0.29	2015 (19)	23,529	0.67
75–79	26,644 (11)	26,431	8657	0.33	1809 (21)	20,834	0.79
≥ 80	41,889 (17)	40,478	14,254	0.35	3084 (22)	37,277	0.92
**Education**							
University degree or higher	58,569 (24)	58,292	12,378	0.21	2395 (19)	31,232	0.54
Certificate or vocational education	155,309 (63)	153,930	40,426	0.26	7556 (19)	95,465	0.62
No certificate	26,824(11)	26,447	9424	0.36	1849 (20)	19,970	0.76
Missing	3597(1.5)	3527	1078	0.31	193 (18)	2515	0.71
**Annual household Income**^**c**^							
High	61,321 (25)	61,174	11,917	0.20	2252 (19)	28,887	0.47
Middle	64,662 (27)	64,297	14,739	0.23	2801 (19)	35,768	0.56
Low	67,170 (28)	66,197	21,235	0.32	4183 (20)	49,568	0.75
Unknown	51,146 (21)	50,526	15,415	0.31	2757 (18)	34,959	0.69
**Area of residence**							
Regional/remote area	113,290 (46)	112,373	25,942	0.23	5132 (20)	67,442	0.60
Major city	126,392 (52)	125,241	36,317	0.29	6679 (18)	79,168	0.63
Missing	4617 (1.9)	4581	1047	0.23	182 (17)	2572	0.56
**History of chronic diseases**							
Asthma	30,608 (13)	30,333	14,635	0.48	2522 (17)	21,914	0.72
COPD	3540 (1.5)	3272	4702	1.44	810 (17)	4034	1.23
Cancer	16,355 (6.7)	15,583	5511	0.35	1265 (23)	14,942	0.96
Diabetes Mellitus	18,730 (7.7)	18,277	7719	0.42	1716 (22)	19,430	1.06
Choric Kidney Diseases	4532 (1.9)	4127	2381	0.58	632 (27)	7683	1.86
Cardiovascular diseases ^d^	13,213 (5.4)	12,671	5537	0.44	1182 (21)	12,902	1.02
**Residence in LTCF**^**e**^							
No	236,911 (97)	235,660	59,853	0.25	11,148 (19)	138,282	0.59
Yes	7388 (3.0)	6535	3453	0.53	845 (25)	10,900	1.68

^a^: per person-year

^b^: a watch/reserve prescription was defined as “related to a test” if there is microbiology testing within 14 days prior to or 7 days after the prescription (see methods)

^c^: It is household income at baseline. Low: < 30,000 AUD, middle: 30000- < 70,000 AUD, high: ≥70,000 AUD

^d^: Included ischemic heart diseases and stroke

^e^: LTCF: Long Term Care Facilities

**Fig. 1:**
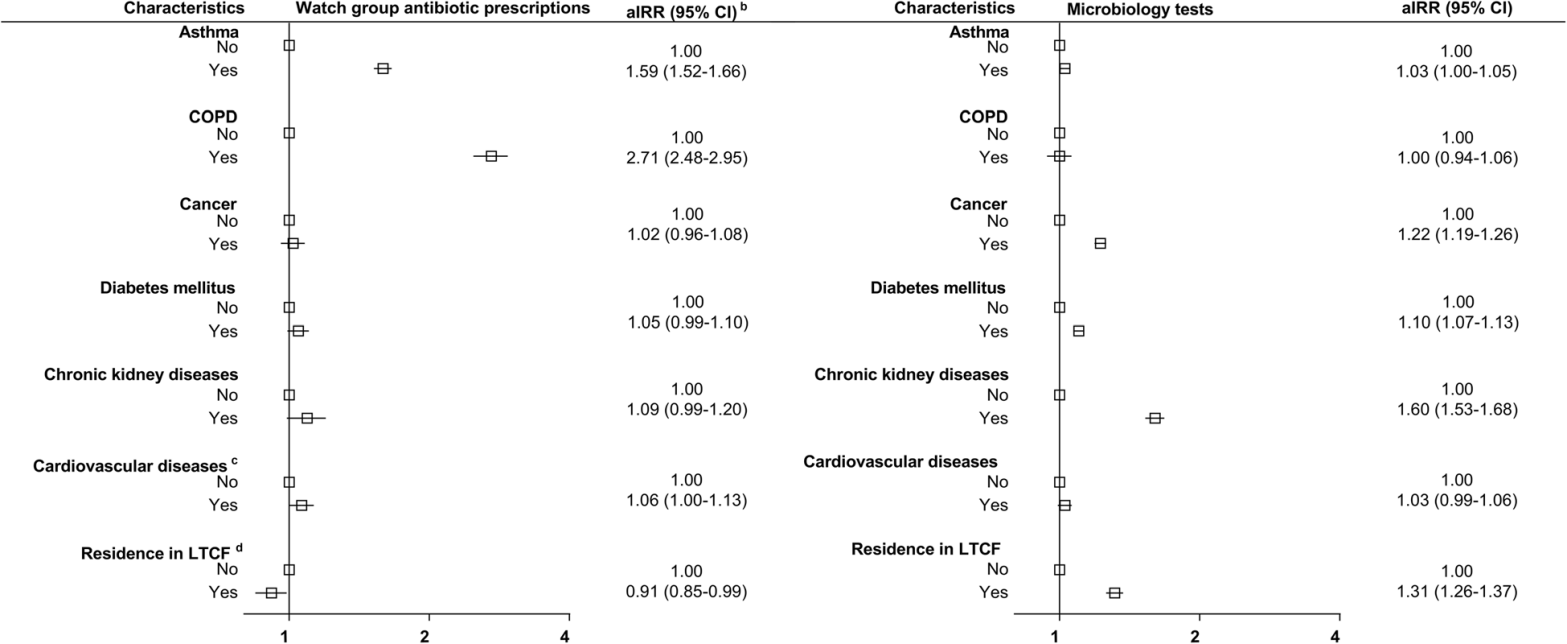
Incidence rate ratio ^a^ for watch group antibiotic dispensing and microbiology testing by participants’ characteristics. **a**: Zero-inflated negative binomial regression adjusted by sex, age, education level, income level, residential remoteness, residence in Long Term Care Facilities (LTCF), history of chronic diseases, number of GP visits in the year before the index date, number of hospital admissions in the year before the index date. **b**: aIRR: adjusted incidence rate ratio, CI: confidence intervals. **c**: Included ischemic heart diseases and stroke. **d**: LTCF: Long Term Care Facilities

After adjustment in multivariable analysis (Fig. 1 and Supplementary Table 3), cancer, diabetes, and chronic kidney diseases were not associated with watch group antibiotic dispensing but associated with a higher likelihood of microbiology testing. Cardiovascular diseases were not associated with watch group antibiotic dispensing nor microbiology testing. The pattern of antibiotic dispensing and microbiology testing in COPD and asthma patients was different from patients with other chronic diseases. While both were strongly associated with greater use of watch group antibiotics, (COPD IRR: 2.71, 95%CI: 2.48–2.95, asthma IRR:1.59, 95%CI: 1.52–1.66), there was almost no increase in microbiology testing in COPD (aIRR:1.00, 95%CI: 0.94–1.06) and asthma (aIRR:1.03, 95%CI: 1.00–1.05) patients, if compared with people without COPD (asthma). Besides, in comparison with people not living in LTCF, those living in LTCF had a lower likelihood of receiving watch group prescriptions (aIRR: 0.91. 95%CI: 0.85–0.99) but a higher likelihood of microbiology testing (aIRR: 1.31 95%CI: 1.26–1.37).

Supplementary Table 4 shows that the incidence rate was 0.28 per person-year for amoxicillin-clavulanate dispensing. After adjustment, COPD, asthma, cancer, diabetes, and chronic kidney diseases, were all significantly associated with a higher likelihood of amoxicillin-clavulanate dispensing. When we restricted watch group antibiotic prescriptions to those without antibiotic prescriptions in the 14 days prior, we found that 39,088 (62%) prescriptions did not follow prior antibiotic use, and the association of chronic respiratory diseases with watch group antibiotic dispensing did not substantially change (see Supplementary Table 5). The incidence rate ratios for certain classes of watch group antibiotic dispensing and microbiology testing by type of test are shown in Supplementary Table 6 and 7, respectively.

Given the strong association of chronic respiratory diseases with watch group antibiotic dispensing, we further examined the relationships according to disease severity (Supplementary Table 8). We divided the population into four groups: 1) no asthma or COPD history; 2) only asthma; 3) less severe COPD (COPD hospitalization < 2 times in the past 3 years); and 4) more severe COPD (COPD hospitalization ≥2 times in the past 3 years). The test for linear trend showed a significant increase in the likelihood of watch group antibiotic dispensing by disease severity (*P* < 0.001), but no increase in microbiology testing (*P* = 0.161), which is consistent with the main analysis.

## Discussion

We found that in a large community-based cohort of older people, there were 26 prescriptions of watch group antibiotics dispensed and 62 microbiology tests for bacterial infections performed per 100 people in 2015. Only 19% of watch group antibiotic prescriptions were accompanied by microbiology testing within − 14 to + 7 days. The patterns of antibiotic dispensing and microbiology testing varied in patients with different chronic health conditions after adjustment. Patients with cancer, diabetes, and chronic kidney diseases did not have a higher likelihood of receiving watch group antibiotics but had a higher likelihood of microbiology testing and receiving amoxicillin-clavulanate. We found people with chronic respiratory diseases, i.e. asthma and COPD, were significantly more likely to receive watch group antibiotics as well as amoxicillin-clavulanate; however, they did not have a comparably higher likelihood of receiving microbiology testing. People in LTCF were not dispensed more watch group antibiotics than those not in LTCF but were more likely to be tested.

Although surveillance programs for antibiotic resistance worldwide [20, 21] are constantly monitoring antibiotic consumption in the population, there are limited data on the appropriateness of antibiotic use. Simply looking at antibiotic consumption may not be enough for fully understanding the factors driving antibiotic resistance. The use of microbiology testing can be considered as a proxy for assessing the appropriateness of antibiotic use [22]. But few studies have examined the rate of microbiology testing in antibiotic treatment. It is only known that empirical antibiotic treatment for infections is quite common in primary care: a study in Europe found that the proportion of empirical antibiotics prescribed for urinary tract infection ranged from 59.4% in the Netherlands to 95.1% in England [23].

When we compared the likelihood of watch group antibiotic dispensing and microbiology testing in the study population, there was a unique pattern identified among people with asthma and COPD, i.e. the high likelihood of watch group antibiotics and amoxicillin-clavulanate dispensing did not accompany a comparably high likelihood of microbiology testing. Meanwhile, these people had a lower proportion of antibiotic prescriptions related to testing. Since the discord was not observed in other patients with chronic diseases, we cannot simply interpret it as the result of susceptibility to infections or a higher likelihood of GP visits. A possible explanation is that the discord might be the result of excessive watch group antibiotic use for exacerbation of chronic respiratory diseases. On the one hand, routine use of antibiotics for asthma exacerbation is not supported by sufficient evidence [24] but is common in clinical practice according to studies in the US and Europe [25–27]. On the other hand, although long-term macrolide use can effectively reduce the exacerbation of COPD due to its anti-inflammatory effect [28], it will significantly increase the emergence of macrolide resistance [29]. Currently, there is no agreement on the use of long-term macrolides or clear suggestions about the monitoring of antibiotic resistance in COPD guidelines [10, 30, 31]. A previous study found that 38% of antibiotic use for COPD in hospitals might be inappropriate [32], which was in line with our results in the community setting. Taken together, our findings support more comprehensive guidelines and stewardship among those with chronic respiratory diseases, which requires identification of barriers to appropriate prescribing and potential mechanisms for monitoring of antibiotic use in these populations. Future strategies may also include the establishment of clear and detailed criteria for selecting patients who are suitable for macrolide prophylaxis in COPD guidelines, [33] introduction of point-of-care testing of C-reactive protein, which has been shown to reduce antibiotic use among patients with COPD exacerbations by identifying those unlikely to benefit from antibiotic therapy [34], and the use of novel macrolides with anti-inflammatory effects but no antibiotic effects which could reduce COPD exacerbations but not lead to increases in antibiotic resistance [35].

Earlier studies reported that inappropriate antibiotic prescribing was common among those living in LTCF [36, 37]. Our study demonstrated that after adjusting for age and comorbidities, the use of watch group antibiotics and amoxicillin-clavulanate was not significantly elevated in this group compared to those living outside of LTCF; however, the likelihood of microbiology testing was higher. The high likelihood of microbiology testing may be the result of over-investigation for asymptomatic bacteriuria, which frequently occurs in LTCF residents [38].

A major strength of our study is the use of data linkage of routinely collected administrative health data. To our knowledge, this approach is underused in studies investigating antimicrobial stewardship. A limitation of our study was the lack of clinical information to determine indications for antibiotic use and the results of microbiology testing. Thus, we were unable to assess the actual appropriateness of each prescription, and whether its temporally related microbiology test was truly in the same episode. Our comparison between rates of antibiotic dispensing and microbiology testing is a crude measure of antibiotic stewardship and should be considered alongside other measures of appropriate antibiotic prescribing. Besides, the MBS database only records the three most expensive pathology items for one patient if there are more than three tests during the one episode (1 day) [39]. This issue will inevitably result in potential under-ascertainment of testing in those episodes with three or more testing records. We used a previously published method [40] to estimate the scale of under-ascertainment from potential incomplete records and found that it would only affect about 11% of all episodes. Therefore, this is unlikely to have a major impact on our findings.

## Conclusions

Watch group antibiotics are commonly dispensed among older adults in the community. This is particularly true for patients with asthma and COPD; however, their likelihood of receiving microbiology testing is not comparably high, indicating the potential for excessive empirical watch group antibiotic use. Since watch group antibiotics have high resistance potential, focusing antibiotic stewardship efforts might be needed among older populations with chronic respiratory diseases in the primary care setting.

## Supplementary information

**Additional File 1 Table S1.** Classification for watch group and reserve group according to the WHO Model List of Essential Medicines

**Additional File 2 Table S2.** Medicare Benefits Schedule (MBS) codes used for GP consultations, aged care facilities (or Long-Term Care Facilities, LTCF) attendance and microbiology testing

**Additional File 3 Table S3.** Incidence rate ratio for watch group antibiotic dispensing and microbiology testing by participants’ characteristics

**Additional File 4 Table S4.** Incidence and incidence rate ratio for dispensed amoxicillin-clavulanate prescriptions by participants’ characteristics

**Additional File 5 Table S5.** Incidence and incidence rate ratio for dispensed watch group antibiotic prescriptions without antibiotic use in the 14 days prior to dispensing by participants’ characteristics

**Additional File 6 Table S6.** Incidence rate ratio for dispensed macrolides and other watch group antibiotics prescriptions by participants’ characteristics

**Additional File 7 Table S7.** Incidence rate ratio for certain types of microbiology testing among 244,299 participants by participants’ characteristics

**Additional File 8 Table S8.** Incidence of dispensed antibiotic prescriptions and microbiology tests and their association between chronic lower respiratory tract diseases

**Additional File 9 Figure S1.** The distribution of the intervals between dispensed script of watch group antibiotics and its closest microbiology test (only include intervals ≤30 days).

## Data Availability

The data that support the findings of this study are available from the Sax Institute but restrictions apply to the availability of these data, which were used under license for the current study, and so are not publicly available. Data are however available from the authors upon reasonable request and with permission of the Sax Institute.

## Abbreviations

WHO: World Health Organization
LTCF: Long-Term Care Facilities
NSW: New South Wales
DHS: Department of Human Services
PBS: Pharmaceutical Benefits Scheme
MBS: Medicare Benefits Schedule
APDC: NSW Admitted Patient Data Collection
RBDM: The NSW Registry of Births, Deaths and Marriages
CHeReL: Centre for Health Record Linkage
ATC: Anatomical Therapeutic Chemical
GP: General practitioner
ICD-10-AM: International Classification of Diseases version 10 Australian Modification
COPD: Chronic obstructive pulmonary disease
aIRR: Adjusted incidence rate ratios
CI: Confidence intervals
