# Supplementary material for: Microbiology testing associated with antibiotic dispensing in older community-dwelling adults
Source: BMC Infect Dis. 2020 Apr 25;20:306. doi: 10.1186/s12879-020-05029-z (PMC7183691; doi:10.1186/s12879-020-05029-z)
Supplement: Supplementary file 1 — Additional File 1 Table S1. Classification for watch group and reserve group according to the WHO Model List of Essential Medicines [file 12879_2020_5029_MOESM1_ESM.docx]

Supplementary Table 1. Classification for watch group and reserve group according to the WHO Model List of Essential Medicines

| **Watch group antibiotics** | |
| --- | --- |
| **Antibiotic class** | **ATC code** |
| Macrolides | J01FA |
| Quinolones and fluoroquinolones | J01M |
| 3rd-generation cephalosporins | J01DD |
| Glycopeptides | J01XA |
| Antipseudomonal penicillins + beta-lactamase inhibitor | J01CR03, J01CR05 |
| Carbapenems | J01DH |
| Penems | J01DI03 |
| **Reserve group antibiotics** | |
| **Antibiotic class** | **ATC code** |
| Aztreonam | J01DF01 |
| 4th & 5th generation cephalosporins | J01DE, J01DI01, J01DI02, J01DI54 |
| Polymyxins | J01XB |
| Fosfomycins | J01XX01 |
| Oxazolidinones | J01XX08, J01XX11 |
| Tigecycline | J01AA12 |
| Daptomycin | J01XX09 |
