# Supplementary material for: Microbiology testing associated with antibiotic dispensing in older community-dwelling adults
Source: BMC Infect Dis. 2020 Apr 25;20:306. doi: 10.1186/s12879-020-05029-z (PMC7183691; doi:10.1186/s12879-020-05029-z)
Supplement: Supplementary file 2 — Additional File 2 Table S2. Medicare Benefits Schedule (MBS) codes used for GP consultations, aged care facilities (or Long-Term Care Facilities, LTCF) attendance and microbiology testing [file 12879_2020_5029_MOESM2_ESM.docx]

Supplementary Table 2. Medicare Benefits Schedule (MBS) codes used for GP consultations, aged care facilities (or Long-Term Care Facilities, LTCF) attendance and microbiology testing

| **GP consultations** | |
| --- | --- |
| **MBS item number** | **Description** |
| 3 to 51 | Attendances by General Practitioners |
| 193,195,197,199, 597, 599 | Attendances by General Practitioners |
| 2497 to 2559 | Attendances by General Practitioners |
| 5000-5067 | Attendances by General Practitioners |
| **Participants aged care facility attendance** | |
| **MBS item number** | **Description** |
| 20, 35, 43, 51, 92, 93, 95, 96, 5010, 5028, 5049, 5067, 5260, 5263, 5265, 5267 | Residential Aged Care Facility Attendances |
| 731 | Contribution to a Multidisciplinary Care Plan, or to a review of a multidisciplinary care plan, for a resident in an aged care facility |
| 903 | Residential Medication Management Review |
| 2125, 2138, 2179, 2220 | Medical practitioner telehealth attendances at a residential aged care facility |
| 10947, 10948, | At the time of the attendance, is located at a residential aged care facility |
| 73934, 73935, | Approved pathology authority from in a residential aged care home or institution |
| 10984 | A care recipient receiving care in a residential aged care service |
| 82223, 82224, 82225 | Telehealth attendance at a residential aged care facility |
| **Microbiology testing** | |
| **MBS item number** | **Description** |
| 69300 | Microscopy of wet film material other than blood, from 1 or more sites, obtained directly from a patient (not cultures) |
| 69303 | Culture and (if performed) microscopy to detect pathogenic micro-organisms from nasal swabs, throat swabs, eye swabs and ear swabs (excluding swabs taken for epidemiological surveillance) |
| 69306 | Microscopy and culture to detect pathogenic micro-organisms from skin or other superficial sites |
| 69312 | Microscopy and culture to detect pathogenic micro-organisms from urethra, vagina, cervix or rectum (except for faecal pathogens) |
| 69316, 69317, | Detection of Chlamydia trachomatis by any method |
| 69318, 69319, | Microscopy and culture to detect pathogenic micro-organisms from specimens of sputum (except when part of items 69324, 69327 and 69330) |
| 69321 | Microscopy and culture of post-operative wounds, aspirates of body cavities, synovial fluid, CSF or operative or biopsy specimens, for the presence of pathogenic micro-organisms involving aerobic and anaerobic cultures and the use of different culture media |
| 69324, 69325, 69327, 69328, 69330, 69331 | Microscopy (with appropriate stains) and culture for mycobacteria - 1 specimen of sputum, urine, or other body fluid or 1 operative or biopsy specimen |
| 69333 | Urine examination (including serial examination) by any means other than simple culture by dip slide |
| 69345 | Culture and (if performed) microscopy without concentration techniques of faeces for faecal pathogens, using at least 2 selective or enrichment media and culture in at least 2 different atmospheres |
| 69354, 69357, 69360, | Blood culture for pathogenic micro-organisms (other than viruses) |
| 69363 | Detection of Clostridium difficile or Clostridium difficile toxin (except if a service described in item 69345 has been performed) |
| 69384, 69387, 69390, 69393, 69396, 69400, 69401 | Quantitation of 1 antibody to microbial antigens not elsewhere described in the Schedule |
| 69471 | Test of cell-mediated immune response in blood for the detection of latent tuberculosis by interferon gamma release assay (IGRA) in the following people |
| 69494, 69495, 69496, 69497, 69498 | Detection of a virus or microbial antigen or microbial nucleic acid (not elsewhere specified) |
