# Supplementary material for: Microbiology testing associated with antibiotic dispensing in older community-dwelling adults
Source: BMC Infect Dis. 2020 Apr 25;20:306. doi: 10.1186/s12879-020-05029-z (PMC7183691; doi:10.1186/s12879-020-05029-z)
Supplement: Supplementary file 4 — Additional File 4 Table S4. Incidence and incidence rate ratio for dispensed amoxicillin-clavulanate prescriptions by participants’ characteristics [file 12879_2020_5029_MOESM4_ESM.docx]

Supplementary Table 4. Incidence and incidence rate ratio ^a^ for dispensed amoxicillin-clavulanate prescriptions by participants’ characteristics

| Variable | Amoxicillin-clavulanate prescriptions | | | |
| --- | --- | --- | --- | --- |
|  | No. | Incidence | aIRR (95%CI) ^b^ | P value |
| **Total** | 67,735 | 0.28 |  |  |
| **Asthma** |  |  |  |  |
| No | 53,584 | 0.25 | 1.00 |  |
| Yes | 14,151 | 0.47 | 1.42 (1.36-1.48) | <0.001 |
| **COPD** |  |  |  |  |
| No | 64,011 | 0.27 | 1.00 |  |
| Yes | 3724 | 1.14 | 2.13 (1.95-2.32) | <0.001 |
| **Cancer** |  |  |  |  |
| No | 61,521 | 0.27 | 1.00 |  |
| Yes | 6214 | 0.40 | 1.10 (1.04-1.16) | <0.001 |
| **Diabetes Mellitus** |  |  |  |  |
| No | 59,143 | 0.26 | 1.00 |  |
| Yes | 8592 | 0.47 | 1.10 (1.05-1.16) | <0.001 |
| **Chronic kidney diseases** |  |  |  |  |
| No | 65,048 | 0.27 | 1.00 |  |
| Yes | 2687 | 0.65 | 1.25 (1.14-1.38) | <0.001 |
| **Cardiovascular diseases ^c^** |  |  |  |  |
| No | 62,007 | 0.27 | 1.00 |  |
| Yes | 5728 | 0.45 | 1.03 (0.97-1.09) | 0.399 |
| **Residence in LTCF ^d^** |  |  |  |  |
| No | 64,389 | 0.27 | 1.00 |  |
| Yes | 3346 | 0.51 | 1.06 (0.98-1.14) | 0.148 |

a: Zero-inflated negative binomial regression adjusted by sex, age, education level, income level, residential remoteness, residence in LTCF, history of chronic diseases, number of GP visits in the year before the index date, number of hospital admissions in the year before the index date

b: aIRR: adjusted incidence rate ratio CI: confidence intervals

c: Included ischemic heart diseases and stroke

d: LTCF: Long Term Care Facilities
