# Supplementary material for: Microbiology testing associated with antibiotic dispensing in older community-dwelling adults
Source: BMC Infect Dis. 2020 Apr 25;20:306. doi: 10.1186/s12879-020-05029-z (PMC7183691; doi:10.1186/s12879-020-05029-z)
Supplement: Supplementary file 8 — Additional File 8 Table S8. Incidence of dispensed antibiotic prescriptions and microbiology tests and their association between chronic lower respiratory tract diseases [file 12879_2020_5029_MOESM8_ESM.docx]

Supplementary Table 8. Incidence of dispensed antibiotic prescriptions and microbiology tests and their association ^a^ between chronic lower respiratory tract diseases

|  | No asthma /COPD | Asthma & No COPD | | Less severe COPD ^c^ | | More severe COPD ^d^ | |  |
| --- | --- | --- | --- | --- | --- | --- | --- | --- |
| **N (%)** | 211,343 (87) | 29,416 (12) | | 2575 (1.1) | | 965 (0.4) | |  |
| **Incidence (person-years)** |  |  | |  | |  | |  |
| Watch group antibiotics | 0.22 | 0.43 | | 1.11 | | 2.37 | |  |
| Amoxicillin-clavulanate | 0.57 | 0.67 | | 1.14 | | 1.37 | |  |
| Microbiology tests | 0.60 | 0.70 | | 1.17 | | 1.40 | |  |
| **Association** | Reference | aIRR (95% CI) ^b^ | P value | aIRR (95%CI) | P value | aIRR (95%CI) | P value | P value for trend |
| Watch group antibiotics | 1.00 | 1.59 (1.52-1.66) | <0.001 | 2.53 (2.29-2.81) | <0.001 | 5.15 (4.43-5.98) | <0.001 | <0.001 |
| Macrolides | 1.00 | 1.66 (1.58-1.74) | <0.001 | 2.60 (2.33-2.91) | <0.001 | 5.83 (4.96-6.85) | <0.001 | <0.001 |
| Other watch group antibiotics | 1.00 | 1.11 (0.97-1.28) | <0.001 | 1.97 (1.55-2.50) | <0.001 | 2.09 (1.55-2.82) | <0.001 | <0.001 |
| Amoxicillin-clavulanate | 1.00 | 1.43 (1.36-1.49) | <0.001 | 2.11 (1.91-2.34) | <0.001 | 3.27 (2.81-3.81) | <0.001 | <0.001 |
| Microbiology tests | 1.00 | 1.02 (1.00-1.05) | 0.079 | 1.00 (0.94-1.07) | 0.998 | 1.03 (0.93-1.14) | 0.622 | 0.161 |

a: Zero-inflated negative binomial regression, adjusted by sex, age, education level, income level, residential remoteness, residence in Long Term Care Facilities (LTCF), history of chronic diseases, number of GP visits in the year before the index date, number of hospital admissions in the year before the index date

b: aIRR: adjusted incidence relative risk; CI: confidence intervals

c: hospitalization <2 times in the past three years

d: hospitalization ≥2 times in the past three years
