# Supplementary material for: Microbiology testing associated with antibiotic dispensing in older community-dwelling adults
Source: BMC Infect Dis. 2020 Apr 25;20:306. doi: 10.1186/s12879-020-05029-z (PMC7183691; doi:10.1186/s12879-020-05029-z)
Supplement: Supplementary file 9 — Additional File 9 Figure S1. The distribution of the intervals between dispensed script of watch group antibiotics and its closest microbiology test (only include intervals ≤30 days). [file 12879_2020_5029_MOESM9_ESM.docx]

Supplementary Figure 1. The distribution of the intervals between a dispensed script of watch group antibiotics and its closest microbiology test (only include intervals ≤ 30 days).
